# Supplementary material for: Seminoma and Embryonal Carcinoma Footprints Identified by Analysis of Integrated Genome-Wide Epigenetic and Expression Profiles of Germ Cell Cancer Cell Lines
Source: PLoS One. 2014 Jun 2;9(6):e98330. doi: 10.1371/journal.pone.0098330 (PMC4041891; doi:10.1371/journal.pone.0098330)
Supplement: File S1 — ZIP file containing DMRforPairs output for significant regions. Please start from the html files. (ZIP) [file pone.0098330.s008.zip › figures/19676.pdf]

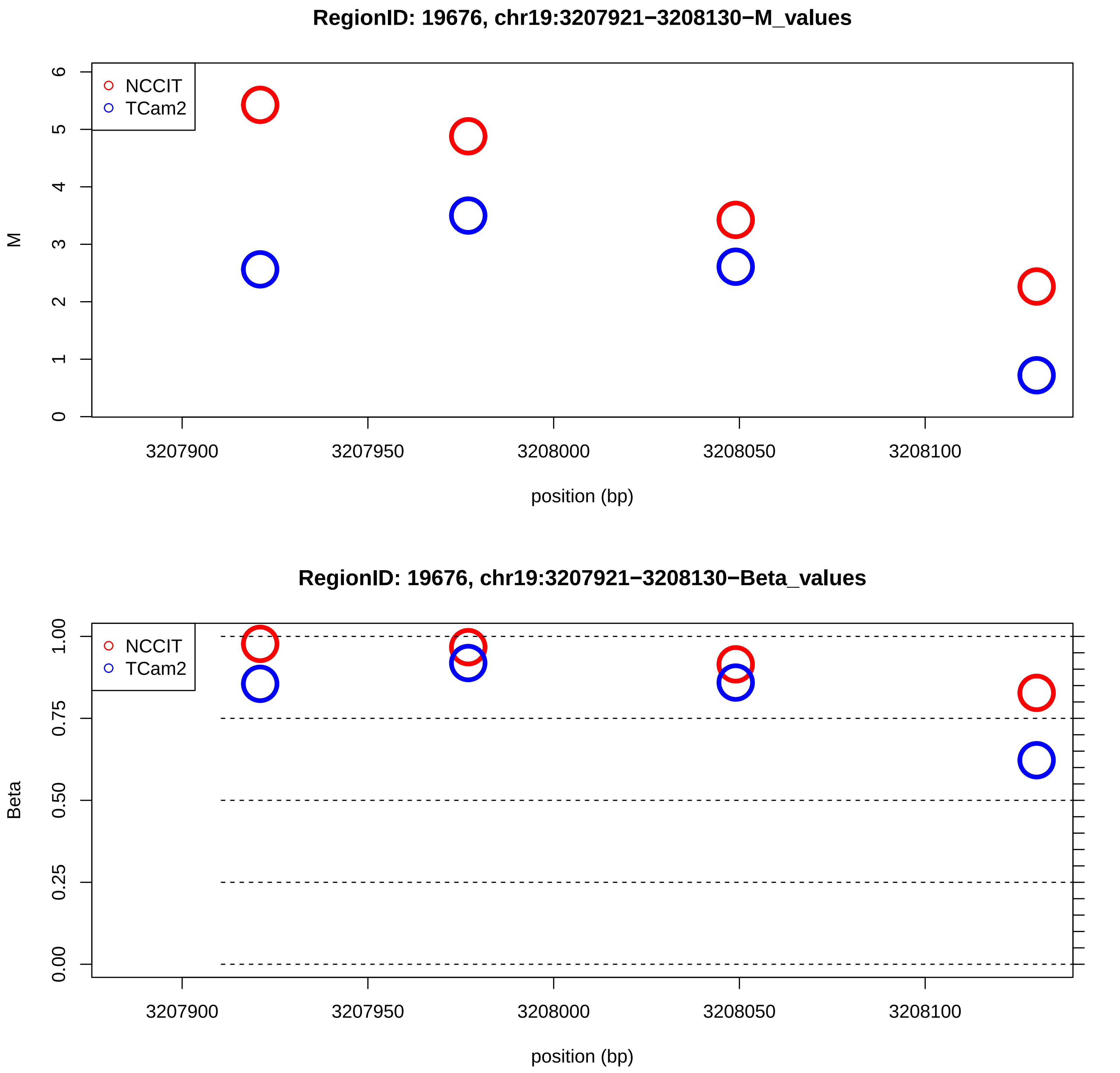
RegionID: 19676, chr19:3207921−3208130−M_values
NCCIT
TCam2
M
0
1
2
3
4
5
6
3207900
3207950
3208000
3208050
3208100
position (bp)
RegionID: 19676, chr19:3207921−3208130−Beta_values
NCCIT
TCam2
Beta
0.00
0.25
0.50
0.75
1.00
3207900
3207950
3208000
3208050
3208100
position (bp)
